# Sociodemographic Drivers of Delays in Seeking Medical Care in the All of Us Cohort

**DOI:** 10.3390/nursrep16020051

**Published:** 2026-02-02

**Authors:** Tadesse M. Abegaz, Efrata Ashuro Shegena, Gabriel Frietze, Muktar Ahmed

**Affiliations:** 1 School of Pharmacy, University of Texas at El Paso, 1101 N Campbell St, El Paso, TX 79902, USA; gafrietze@utep.edu; 2 Division of Pharmaceutical Evaluation and Policy, College of Pharmacy, University of Arkansas for Medical Sciences, 4301 W Markham St, Little Rock, AR 72205, USA; eashegena@uams.edu; 3 College of Medicine and Public Health, Flinders University, Bedford Park, Adelaide, SA 5042, Australia; muktar.ahmed@mymail.unisa.edu.au

**Keywords:** medical care, nursing, All of Us cohort, access, utilization

## Abstract

**Background/Objectives**: This study examined the reasons and sociodemographic drivers behind delays in seeking medical care among participants in the All of Us Research Program. **Methods**: A cross-sectional study was conducted using data collected between 2018 and 2024. The primary outcome was the prevalence of reasons for delayed medical care (DMC). Descriptive statistics were used to calculate the prevalence of the various reported reasons for delayed medical care. Binary logistic regression was applied to examine the association between sociodemographic characteristics and each reported reason for delayed medical care. **Results**: Out of a total of 633,000 All of Us participants, 300,820 participants had complete data on the healthcare utilization and access survey and were eligible for final analysis. The most common reported reasons for DMC were out-of-pocket expenses (16.68%), nervousness about seeing a provider (14.18%), and inability to get time off work (11.04%). Females had significantly higher odds of DMC due to out-of-pocket costs (OR = 1.31, 95% CI: 1.28–1.33). Black (OR = 0.81, 95% CI: 0.78–0.84) and Asian (OR = 0.94, 95% CI: 0.89–0.99) individuals had lower odds of DMC due to out-of-pocket costs. Married individuals had more than twice the odds of DMC due to childcare responsibilities (OR = 2.45, 95% CI: 2.33–2.56). **Conclusions**: A significant proportion of participants reported DMC due to various reasons, with financial, medical visit anxiety, and work-related reasons being the most common. These findings highlight actionable intervention targets, including nurse-led cost navigation and financial counseling, flexible scheduling/telehealth to reduce work-related delays, and patient-centered communication and outreach strategies to reduce visit-related anxiety and support caregiving and transportation needs.

## 1. Introduction

The United States (U.S.) healthcare system is characterized by a mixed public–private insurance structure, with coverage obtained through employer-sponsored plans, public programs such as Medicare and Medicaid, or the individual market. Unlike systems with universal coverage, access to care in the U.S. is often mediated by insurance status, benefit design, and cost-sharing mechanisms such as deductibles, copayments, and coinsurance [1]. Structural and organizational characteristics of the U.S. healthcare system may affect individuals’ access to timely medical care.

Delayed medical care (DMC) refers to the postponement or avoidance of accessing a healthcare service despite the presence of symptoms or scheduled appointments [2]. National and regional cross-sectional surveys have shown that DMC is widespread in the U.S., ranging between 33% and 38% [3]. Delays in care have been shown to negatively affect patient outcomes and health system performance, including higher emergency room utilization and lower patient satisfaction [4]. Survey-based studies have identified key explanations that have been reported as reasons for postponement or avoidance of care, including high deductibles, copayments, and out-of-pocket costs, preferences for self-care or alternative treatments, distrust of healthcare providers, fear of diagnosis or medical procedures, and concerns related to time and cost [5,6]. Other studies have revealed that living in rural areas, transportation issues, work-related obligations, long waiting times, and childcare responsibilities were linked with DMC [7,8,9,10,11,12].

Despite several reports on the adverse health outcomes of DMC, significant gaps remain in the understanding of the broad scope of DMC and its sociodemographic determinants within the large and diverse contemporary U.S. population. Existing studies have primarily focused on small samples with limited exploration of the broader contributing factors in DMC [13]. The most recent version of the All of Us (AoU) research program data includes over 633,000 participants, allowing for more robust analyses across historically underrepresented groups. Therefore, the present study used data from the AoU, a nationwide cohort of over half a million U.S. participants, to (1) quantify the prevalence of delayed medical care and (2) identify the primary reasons and sociodemographic factors underlying DMC. Findings from this work will inform health systems and policymakers on the key drivers of timely care and provide a basis for developing patient-centered interventions to reduce DMC. In addition, the findings are directly relevant to healthcare practitioners, including nurses, who frequently serve as the first point of contact for patients navigating access barriers, including cost concerns, appointment anxiety, transportation challenges, and competing caregiving or work responsibilities. Particularly, nurses assess unmet needs, provide patient education and counseling, coordinate referrals, and implement culturally responsive communication strategies that can reduce delays and improve the timely utilization of preventive and chronic care services. Therefore, the current study can help nursing teams identify groups at higher risk of DMC and inform the development of nurse-led interventions.

## 2. Materials and Methods

### 2.1. Study Design and Data Source

This study is a cross-sectional analysis of data from the National Institutes of Health AoU Research Program, a large, ongoing, longitudinal cohort designed to advance precision medicine and improve health outcomes through the inclusion of diverse populations across the United States. The AoU program was launched nationally in May 2018 and aims to enroll at least one million adults aged 18 years or older who consent to share health-related data for research purposes. AoU places particular emphasis on recruiting individuals from groups that are historically underrepresented in biomedical research, including racial and ethnic minorities, individuals with lower income or educational attainment, rural residents, and individuals with disabilities [14].

Participants are recruited through a nationwide network of more than 340 enrollment sites, including academic medical centers, federally qualified health centers, the Veterans Health Administration, and community-based partners, as well as through a direct volunteer pathway that allows individuals to enroll online independent of healthcare system affiliation. Enrollment is voluntary, and all participants provide electronic informed consent. Data collection occurs through multiple modalities, including standardized health surveys, electronic health records, physical measurements, biospecimen collection, and optional digital health data from wearable devices. De-identified participant data are made available to approved investigators through the secure, cloud-based All of Us Researcher Workbench, which supports reproducible analyses using open-source software including Python and R programming (https://allofus.nih.gov/ Access on 27 January 2026) [15,16].

### 2.2. All of Us Cohort and Survey Procedures

Following enrollment and completion of the required baseline demographic survey (“The Basics”), participants are invited to complete additional validated surveys that capture lifestyle factors, overall health, medical history, family history, and healthcare access and utilization. The surveys are also administered electronically, are available in English and Spanish, and are written at approximately a fifth-grade reading level to promote accessibility. All survey instruments undergo cognitive testing and validation prior to deployment.

The present study specifically utilizes data from the Healthcare Access and Utilization Survey, which is administered approximately 90 days after enrollment. This survey captures self-reported information on healthcare use and barriers to care, including reasons for delaying or avoiding medical care. These reasons form the primary outcomes of interest in this analysis and are categorized into financial, psychosocial, and travel/work-related delays. Detailed documentation of survey instruments and variables is publicly available through the AoU Researcher Workbench and Data Browser [17].

### 2.3. Sampling Considerations and Potential Selection Bias

Given the voluntary (non-probability) participation in the AoU, findings should be interpreted as associations among AoU survey respondents rather than population-representative estimates for all U.S. adults. The non-probability nature of the selection/enrollment of participants primarily implies reduced generalizability (external validity), while internal comparisons remain informative for identifying patterns of association within this large, diverse cohort. Additionally, selection bias is possible, particularly if individuals who choose to enroll or complete follow-up surveys differ systematically from those who do not. On top of that, completion of the Healthcare Access and Utilization Survey is optional and occurs after baseline enrollment, which may further introduce response bias. However, AoU’s large sample size, broad geographic coverage, and intentional oversampling of populations that are historically underrepresented in biomedical research enhance the diversity and analytic value of the cohort.

The AoU research program intentionally recruits participants from groups that are historically underrepresented in biomedical research (e.g., racial/ethnic minority populations, individuals with lower income or educational attainment, rural residents, and individuals with disabilities). This recruitment emphasis increases sample sizes for subgroup analyses.

### 2.4. Study Variables

The primary outcome variables were the reasons for delayed medical care. Participants were asked: “In the past 12 months, have you delayed getting medical care for the following reasons?” (1) lack of transportation; (2) living in a rural area where the distance to the healthcare provider was too far; (3) feeling nervous about seeing a healthcare provider; (4) inability to get time off work; (5) lack of childcare; (6) responsibility for caring for an adult and being unable to leave them; (7) inability to afford the copay; (8) high deductible or inability to afford it; and (9) having to pay out of pocket for some or all of the procedure. These nine reasons were subsequently grouped into three major subcategories to facilitate analysis: (1) financial reasons, which included inability to afford the copay, high deductible, and out-of-pocket expenses; (2) psychosocial reasons, which included feeling nervous about seeing a healthcare provider, lack of childcare, and elderly care responsibilities; and (3) travel-related reasons, which included rural residence, inability to take time off work, and lack of transportation.

Sociodemographic variables included age, gender, race, ethnicity, income, insurance status, education, and employment. Age in years was categorized as less than 40 years, 40 to 64 years, 65 to 74 years, 75 to 84 years, and 85 years or older. Annual income in dollars was categorized as less than USD 25,000, USD 25,000 to USD 50,000, USD 50,000 to USD 100,000, USD 100,000 to USD 200,000, and more than USD 200,000. Insurance status was categorized as insured versus noninsured. Education was categorized as no high school diploma, high school diploma/GED, and college and greater.

### 2.5. Study Population

Participants who were enrolled in the AoU Research Program between May 2018 and April 2024 were eligible for inclusion. Eligibility criteria for the present analysis included the following: (1) age between 18 and 85 years at the time of survey completion, (2) completion of the baseline demographic survey (“The Basics”), and (3) completion of the Healthcare Access and Utilization Survey with valid responses to items assessing delayed medical care. Participants older than 85 years were excluded to ensure consistency with age categorizations and to reduce sparse data issues in extreme age strata.

Among approximately 633,000 AoU participants who had completed at least one survey during the study period, 305,860 individuals completed the Healthcare Access and Utilization Survey. Participants were further assessed for completeness of data on the primary outcome variables (reasons for delayed medical care) and key sociodemographic covariates. Of the 305,860 participants who completed the Healthcare Access and Utilization Survey, 5040 were excluded due to missing responses on the DMC items and sociodemographic variables, yielding a final analytic sample of 300,820.

### 2.6. Statistical Analysis

Descriptive statistics were used to estimate the prevalence of delay in seeking medical care and to characterize reasons for delay across different sociodemographic groups. Results were presented in tables and graphs where appropriate. Binary logistic regression examined the association between sociodemographic predictors (e.g., age, sex, race/ethnicity, income, insurance status) and reported delays in care. The sociodemographic predictors were identified using Andersen’s behavioral model, a behavioral model that predicts a sequence of predisposing, enabling, and need factors that influence a person’s utilization of health service [18]. Prevalence estimates were presented as unweighted proportions among eligible respondents because survey weights are not available for population-representative inference in the AoU volunteer cohort. In addition to reason-specific models, we fit a primary multivariable logistic regression model with a binary outcome indicating any delayed medical care (yes/no), defined as reporting at least one reason for delayed care in the past 12 months. All statistical tests were 2-sided, with a significant threshold set at the α  = 0.05 level. Analyses were conducted using R software version 4.1.0 (The R Foundation for Statistical Computing) via the NIH AoU Research Workbench. The corresponding analytic notebook is referenced here [19].

## 3. Results

### 3.1. Population Characteristics

A total of 300,820 participants (65.3% female) were included in the final analysis. The age distribution shows that the largest proportion of individuals was in the 40–64-year age group, comprising 43.3%, followed by participants younger than 40 years (26.4%). In terms of race, the study sample was predominantly White, 69.1%. Ethnically, the sample was largely non-Hispanic/Latino, accounting for 86.7% of participants. Education levels within the sample show that the majority of participants had at least a college degree, with 84.8% falling into the category of college education or higher. In terms of marital status, nearly fifty percent of the participants were married (Table 1).

### 3.2. Prevalence of Reported Reasons for DMC in the All of Us Cohort

**Figure 1:**
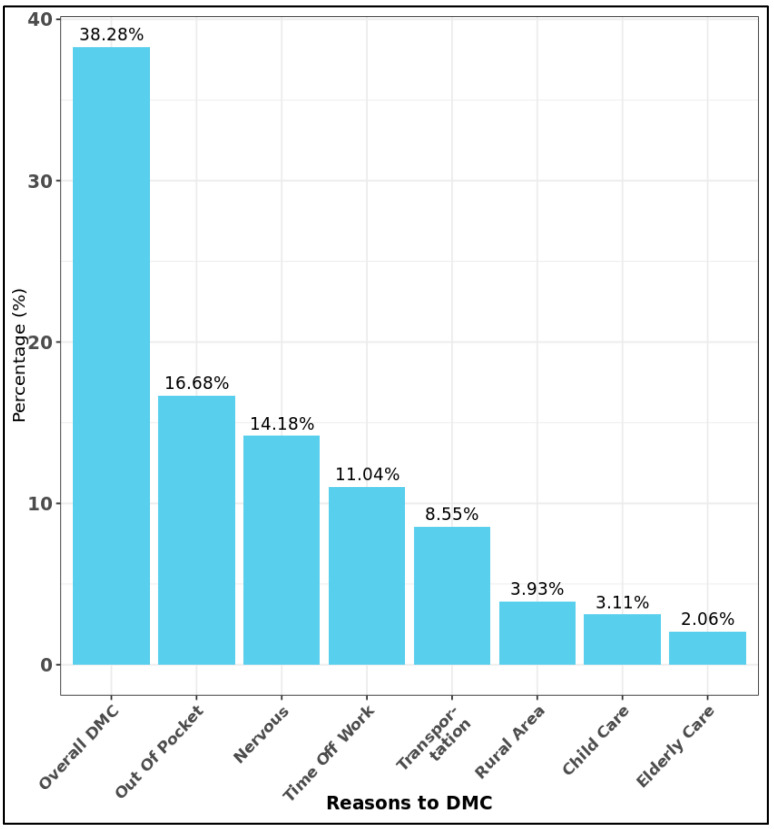
Prevalence of delayed medical care in the All of Us cohort. DMC: Delayed medical care.

The most common reported reasons for DMC were out-of-pocket expenses, nervousness, and time off work, with 50,184 (16.7%) respondents, 42,654 (14.2%) respondents, and 33,223 (11.0%) respondents, respectively. Transportation issues also affected 25,729 (8.6%) respondents, suggesting that travel difficulties remain an important barrier for some participants. In contrast, childcare, rural areas, and elderly care were less common reasons for DMC, with only 9362 (3.1%) respondents, 11,808 (3.9%) respondents, and 6200 (2.1%) respondents, respectively. Because respondents were allowed to select multiple reasons for delayed care, the overall prevalence of DMC due to at least one reason was 38.28%. (Figure 1).

### 3.3. Factors Associated with Overall DMC Due to Any Reason

Delays in seeking medical care for any reason were significantly associated with multiple sociodemographic and socioeconomic factors. Females had higher odds of reporting any delayed care compared with males (OR = 1.44 [1.42–1.47]). Racial and ethnic differences were observed, with Black (OR = 0.90 [0.88–0.93]), Asian (OR = 0.83 [0.79–0.87]), and Hispanic (OR = 0.86 [0.84–0.89]) participants having lower odds of DMC compared with White participants, whereas individuals classified as Other race had slightly higher odds of delay (OR = 1.17 [1.14–1.20]). Income showed a strong inverse association with delayed care. Compared with the lowest-income group, participants earning USD 50,000–USD 100,000 had lower odds of delay (OR = 0.66 [0.64–0.68]), with progressively lower odds among those earning USD 100,000–USD 200,000 (OR = 0.48 [0.47–0.50]) and more than USD 200,000 (OR = 0.49 [0.47–0.50]). Insurance status was also strongly associated with DMC; insured individuals had substantially lower odds of reporting any delay compared with uninsured participants (OR = 0.61 [0.59–0.63]).

Employment status was not meaningfully associated with overall DMC (OR = 1.00 [0.98–1.02]). Educational attainment showed modest associations, with individuals holding a high school diploma (OR = 1.06 [0.99–1.13]) or college education or higher (OR = 1.04 [0.98–1.11]) having slightly higher odds of delay compared with those without a high school diploma. Married participants were less likely to report DMC than unmarried participants (OR = 0.83 [0.81–0.84]). Age demonstrated a strong and graded association, with substantially lower odds of DMC among older adults, including those aged 40–64 years (OR = 0.56 [0.55–0.57]), 65–74 years (OR = 0.19 [0.18–0.19]), 75–84 years (OR = 0.13 [0.12–0.14]), and ≥85 years (OR = 0.12 [0.10–0.13]), compared with participants younger than 40 years (Table 2).

### 3.4. Factors Associated with DMC Due to Financial Reasons

DMC due to copays, deductibles, and out-of-pocket costs was significantly associated with demographic and socioeconomic factors. Females had consistently higher odds of delay compared to males across all cost types: copay (OR = 1.43 [1.39–1.48]), deductible (OR = 1.33 [1.29–1.36]), and out-of-pocket costs (OR = 1.31 [1.28–1.33]). Racial and ethnic minorities generally had lower odds of delay due to financial reasons than White individuals. For copays, Asian (OR = 0.69 [0.64–0.75]) and Hispanic (OR = 0.86 [0.82–0.90]) individuals reported fewer delays; similarly, for deductibles, Black (OR = 0.89 [0.85–0.93]), Asian (OR = 0.77 [0.72–0.83]), and Hispanic (OR = 0.85 [0.81–0.89]) individuals had reduced odds. Out-of-pocket delays were also lower for Black (OR = 0.81 [0.78–0.84]), Asian (OR = 0.94 [0.89–0.99]), and Hispanic (OR = 0.82 [0.79–0.86]) populations.

**Figure 2:**
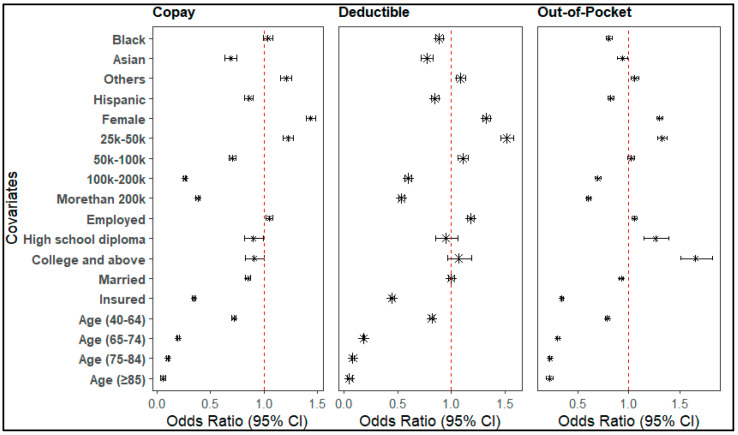
Factors associated with delay in seeking medical care due to financial reasons.

Income demonstrated a strong and consistent association with delayed care: compared to low-income groups, those earning USD 100 k–USD 150 k had lower odds of delay for copays (OR = 0.26 [0.25–0.28]), deductibles (OR = 0.60 [0.57–0.63]), and out-of-pocket costs (OR = 0.70 [0.67–0.73]); individuals earning over USD 200 k showed the lowest odds of delay due to copay (OR = 0.38 [0.36–0.40]), deductible (OR = 0.54 [0.51–0.56]), and out-of-pocket costs (OR = 0.61 [0.58–0.63]). Insurance status had a consistently strong protective effect from all types of financial delays: insured individuals had significantly reduced odds of delay for copay (OR = 0.34 [0.33–0.36]), deductible (OR = 0.45 [0.43–0.47]), and out-of-pocket costs (OR = 0.34 [0.31–0.35]). Employment status showed a modest increase in delay for deductibles (OR = 1.18 [1.15–1.21]) and out-of-pocket costs (OR = 1.06 [1.05–1.08]). Education had mixed effects, in which high school graduates showed slightly reduced odds of copay delay (OR = 0.90 [0.82–0.99]), but higher education was linked to greater delays in out-of-pocket costs (e.g., college degree or above, OR = 1.66 [1.51–1.82]) (Figure 2). Numeric results corresponding to regression figures are provided in Supplementary Table S1.

### 3.5. Factors Associated with DMC Due to Provider and Caregiving Barriers (Psychosocial)

DMC due to psychosocial reasons was significantly associated with various demographic and socioeconomic factors. Compared to White individuals, Black (OR = 0.63, 95% CI: 0.61–0.66), Asian (OR = 0.61, 95% CI: 0.57–0.64), and Hispanic individuals (OR = 0.77, 95% CI: 0.74–0.80) had reduced odds of delaying care due to fear of providers. In contrast, Black (OR = 1.32, 95% CI: 1.22–1.42) and Hispanic (OR = 1.10, 95% CI: 1.03–1.18) individuals had higher odds of delay due to lack of childcare, while Asians had lower odds (OR = 0.73, 95% CI: 0.65–0.83). For elderly care, Black individuals had slightly higher odds (OR = 1.10, 95% CI: 1.01–1.20). Gender differences were consistent across all psychosocial categories: females had significantly increased odds of delay due to fear (OR = 1.53, 95% CI: 1.50–1.57), childcare (OR = 2.50, 95% CI: 2.36–2.65), and elderly care (OR = 1.64, 95% CI: 1.54–1.74).

**Figure 3:**
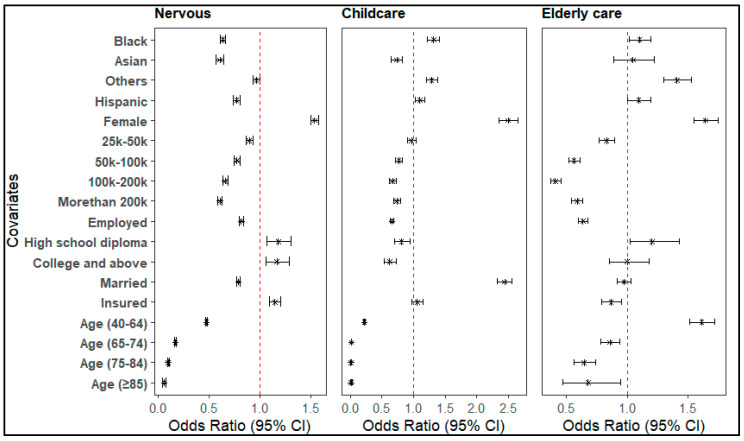
Factors associated with DMC due to provider and caregiving barriers (psychosocial).

Employment showed a significant association with DMC due to psychological reasons. Employment was associated with lower odds of delay due to provider fear (OR = 0.81, 95% CI: 0.79–0.83), childcare (OR = 0.66, 95% CI: 0.63–0.69), and elderly care (OR = 0.64, 95% CI: 0.59–0.67). Married individuals were less likely to DMC due to provider fear (OR = 0.79, 95% CI: 0.77–0.81) and elderly care (OR = 0.87, 95% CI: 0.79–0.96), but had higher odds of delay due to childcare (OR = 2.45, 95% CI: 2.33–2.56). Insurance and age also played critical roles. Insured individuals had increased odds of DMC due to provider fear (OR = 1.15, 95% CI: 1.09–1.20) and elderly care (OR = 1.61, 95% CI: 1.51–1.71), while insurance status was not significantly associated with childcare-related delays (OR = 1.06, 95% CI: 1.00–1.11, *p* = 0.17). Increasing age was consistently associated with reduced odds of delay across all categories. For provider fear, the odds dropped markedly with age: 40–64 years (OR = 0.47, 95% CI: 0.46–0.48), 65–74 (OR = 0.17, 95% CI: 0.16–0.18), 75–84 (OR = 0.10, 95% CI: 0.09–0.10), and 85+ (OR = 0.06, 95% CI: 0.04–0.07). Similar trends were seen for childcare (e.g., 65–74 years: OR = 0.17, 95% CI: 0.16–0.18) and elderly care (65–74: OR = 0.86, 95% CI: 0.78–0.94; 75–84: OR = 0.64, 95% CI: 0.56–0.73; 85+: OR = 0.68, 95% CI: 0.47–0.95) (Figure 3).

### 3.6. Factors Associated with DMC Due to Travel–Related Barriers/Reasons

Most sociodemographic factors were found to be associated with DMC related to distance, time constraints, and transportation. For DMC due to distance, Black (OR = 0.85, 95% CI: 0.80–0.90), Asian (OR = 0.41, 95% CI: 0.35–0.48), and Hispanic (OR = 0.66, 95% CI: 0.61–0.70) individuals had significantly lower odds of delaying care, while females had higher odds (OR = 1.22, 95% CI: 1.17–1.27). Higher income and employment reduced delay odds, with those earning USD 100 k–USD 200 k showing the lowest odds (OR = 0.27, 95% CI: 0.25–0.29). Marital status also lowered DMC risk due to distance, with married individuals less likely to delay (OR = 0.92, 95% CI: 0.88–0.95).

**Figure 4:**
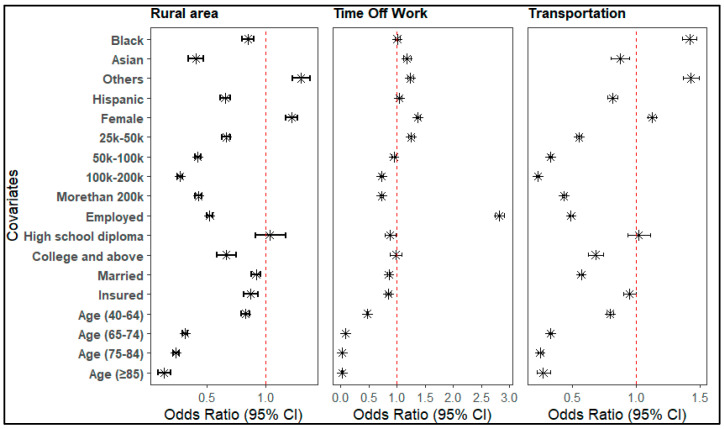
Factors associated with DMC due to travel–related barriers.

In contrast, delayed care due to lack of time off work was associated with higher odds among Asian individuals (OR = 1.18, 95% CI: 1.11–1.25) and females (OR = 1.37, 95% CI: 1.33–1.41), while Hispanic ethnicity showed no significant effect. Employed individuals had much higher odds of delay (OR = 2.82, 95% CI: 2.74–2.90), underscoring time off work as a key barrier. Regarding transportation-related delays, Black individuals were more likely to delay (OR = 1.42, 95% CI: 1.36–1.47), whereas Asian and Hispanic individuals had lower odds. Females had slightly higher odds (OR = 1.13, 95% CI: 1.11–1.16) (Figure 4).

## 4. Discussion

In this large, contemporary analysis of the AoU Research Program, we found that DMC was common and driven by a combination of financial, psychological, and work-related barriers. Approximately one in six participants reported DMC due to out-of-pocket costs, while nervousness about seeing a healthcare provider and inability to obtain time off work were also highly prevalent reasons. Importantly, the factors associated with DMC varied substantially depending on the specific reason for delay, underscoring the multidimensional nature of healthcare access barriers.

Our findings are broadly consistent with prior national estimates of DMC in the U.S. derived from cross-sectional survey data, which identified cost, fear or anxiety related to medical visits, and work-related constraints as leading reasons for DMC [5,6,20]. However, financial barriers such as copayments and deductibles play a less prominent role in DMC in healthcare systems in other countries with universal coverage [21]. Nonetheless, non-financial barriers—including wait times, caregiving responsibilities, and work constraints—continue to influence DMC in universal healthcare settings [22].

Our analysis identifies distinct patterns of association across delay types and various sociodemographic factors, offering a more nuanced understanding of how barriers to care operate. The association between DMC and gender differences was particularly pronounced across nearly all DMC categories. Females consistently had higher odds of delaying care than males, especially for psychosocial reasons such as childcare and elderly care responsibilities. These findings align with prior literature showing that women are more likely to delay or forgo care due to competing caregiving demands and role-related constraints [23]. While women generally utilize healthcare services more frequently than men, this higher utilization may paradoxically increase exposure to structural and logistical barriers, particularly when caregiving responsibilities limit scheduling flexibility. Our results reinforce the need for healthcare delivery models that better accommodate caregiving roles, such as extended clinic hours, telehealth options, and integrated family support services [23].

Racial and ethnic differences in DMC were complex and varied by reason for delay. Overall, Black, Asian, and Hispanic participants were less likely than White participants to report financial-related DMC, a finding that has also been observed in prior AoU-based analyses, including [24]. However, when examining non-financial barriers, Black participants had higher odds of delays related to transportation, childcare, and elderly care [24]. These findings suggest that while financial barriers remain critical, non-cost structural barriers may play a disproportionately important role for certain racial and ethnic groups. This pattern aligns with broader evidence that logistical barriers (transportation access, caregiving demands, scheduling flexibility, and geographic constraints) can meaningfully impede timely care, particularly for marginalized groups, even when cost barriers are reduced [24]. This distinction is important for policy and intervention design, as strategies focused solely on reducing out-of-pocket costs may not adequately address access challenges driven by transportation availability, caregiving responsibilities, or geographic constraints.

Age was strongly and inversely associated with delayed care across most categories. Older participants, particularly those aged 65 years and above, had substantially lower odds of delaying care due to childcare, work-related constraints, and provider-related anxiety. This pattern is consistent with prior studies showing that younger adults are more likely to delay care, often due to employment demands, caregiving responsibilities, and lower insurance stability [25]. In the U.S. context, near-universal Medicare coverage among older adults likely contributes to reduced financial barriers, despite higher overall healthcare utilization in this age group. These findings highlight the importance of age-specific strategies to reduce delays, particularly among working-age adults [26]. As reported in our study, those with insurance status had lower odds of delay in seeking medical care. However, despite insurance coverage, some individuals might delay seeking medical care, especially due to behavioral or attitudinal changes. It was demonstrated in our study that those with insurance coverage were 15% more likely to delay/skip visits due to being nervous about seeing a health care provider compared to uninsured adults. Nervousness may be attributed to feelings of discrimination and negative experiences with health care [27].

Employment status demonstrated a dual role in delayed medical care. While employment may confer financial benefits through employer-sponsored insurance, employed individuals had substantially higher odds of DMC due to a lack of time off work. This finding is consistent with prior evidence showing that limited access to paid leave and inflexible work schedules remain significant barriers to healthcare utilization. Policies that expand paid sick leave and promote workplace flexibility may therefore play a critical role in reducing work-related delays in care [28].

In this study, marital status demonstrated a nuanced association with delays in seeking medical care. Overall, married participants were less likely to report delayed care across most reasons, suggesting that marital partnerships may provide social, logistical, or financial support that facilitates healthcare access. Spousal support may help mitigate barriers such as transportation challenges, appointment coordination, and decision-making, thereby reducing the likelihood of DMC [29]. However, this association did not extend to childcare-related delays. Married individuals had substantially higher odds—more than twofold—of DMC due to childcare responsibilities. This finding likely reflects the increased caregiving demands faced by married households, particularly those with dependent children, especially when a child has an activity limitation, which has been associated with higher rates of delayed or forgone care [30]. When childcare is unavailable or difficult to arrange, individuals may prioritize caregiving responsibilities over their own healthcare needs, resulting in postponed or forgone care. Prior evidence supports this interpretation; cross-sectional studies have shown that childcare barriers are among the most frequently cited reasons for delayed or missed medical care, especially among women [31]. One study reported that more than half of women who delayed care identified childcare as the primary barrier, exceeding transportation and insurance-related obstacles, and participants perceived access to childcare as more difficult than access to healthcare itself [31]. Together, these findings highlight the dual role of marriage as both a promoting factor for healthcare access and a source of competing caregiving demands, underscoring the importance of family-centered interventions, flexible scheduling, and childcare support services to reduce delays in care.

### 4.1. Strengths and Limitations

The current study has several important strengths. Drawing from over 300,000 participants in the demographically diverse AoU Research Program, it provides high statistical power. Additionally, the current study moves beyond traditional financial explanations to investigate a wide range of factors contributing to delayed medical care, including psychological, occupational, caregiving, and logistical barriers. The use of multivariable logistic regression strengthens the analysis by adjusting for confounders and identifying independent factors of DMC. Despite these strengths, this study has some limitations. First of all, the current study did not differentiate the types of healthcare services delayed, which limits contextual understanding. The current study also lacks assessments of the clinical consequences of DMC, including morbidity and mortality. In addition, participation in the AoU cohort is voluntary, and the sample is disproportionately White, insured, and highly educated, which may limit generalizability to the broader U.S. population. Lastly, the current study incorporated an extended data-collection period (2018–2024), which spans major changes in healthcare access and utilization, particularly during the COVID-19 pandemic. Pandemic-related disruptions (e.g., lockdowns and reduced in-person services) could have increased DMC or shifted the distribution of reported reasons for delayed care.

### 4.2. Implications for Nursing Practice and Research

Our findings have several implications for nursing practice and nursing research. First, because the reported reasons for delayed care included out-of-pocket costs, nervousness about seeing a provider, and inability to get time off work, nursing assessments in ambulatory and community settings may benefit from routinely screening for these barriers during intake, triage, and follow-up. Brief, structured questions (e.g., concerns about costs, anxiety/fear related to visits, work schedule conflicts, transportation, and caregiving responsibilities) can help nurses identify patients at risk of DMC. Second, the higher odds of DMC among females—especially for caregiving-related barriers—underscore the need for nurse-led, family-centered care approaches, including flexible scheduling, telehealth/telephone follow-up, and connection to community resources for childcare and caregiver support. Third, the elevated odds of transportation-related delays among Black participants highlight the importance of nursing-driven care coordination strategies such as transportation resource referrals, reminder/recall systems, and community outreach partnerships, particularly for patients at risk of missed or postponed visits. Fourth, work-related delays were strongly associated with employment status, suggesting the potential value of nurse-led workplace-friendly care models (extended clinic hours, asynchronous messaging, and rapid access visits) and patient education on how to navigate sick leave policies and appointment documentation when appropriate. Finally, nursing research should build on these results by testing targeted, nurse-led interventions (e.g., navigation, motivational interviewing to address visit anxiety, resource linkage for cost/transportation, and tailored outreach for high-risk groups) and evaluating outcomes such as appointment completion, time-to-care, patient-reported access, and downstream clinical outcomes.

## 5. Conclusions

DMC was common in this large AoU sample and reflected multiple, co-occurring barriers, most notably out-of-pocket costs, visit-related nervousness, work constraints, transportation, and caregiving demands. The strongest associations differed by DMC reason, indicating that MC is a multidimensional construct that should be studied using reason-specific outcomes rather than a single binary measure. Health systems and nursing teams can translate these findings into practice by implementing routine barrier screening during intake and follow-up, strengthening cost-navigation and benefits counseling, expanding flexible scheduling and telehealth options to reduce work-related delays, and developing referral pathways for transportation and caregiver support. Policies and programs that directly address caregiving burden—particularly among women and married households—may be important leverage points for reducing delayed care. Future research should evaluate targeted, nurse-led, and system-level interventions tailored for specific MDC reasons and assess their impact on timely utilization and downstream health outcomes.

## Figures and Tables

**Table 1:** Sociodemographic characteristics of eligible participants in All of Us Cohort (2018–2024).

Characteristics	n (%)
Gender	
Male	104,379 (34.7)
Female	196,441 (65.3)
Age	
<40	79,315 (26.4)
≥40–64	130,148 (43.3)
≥65–75	64,557 (21.5)
≥75–85	23,998 (8.0)
≥85	2802 (0.9)
Race	
White	207,832 (69.1)
Black	27,846 (9.3)
Asian	10,195 (3.4)
Others	54,947 (18.3)
Ethnicity	
Non-Hispanic/Latino	260,885 (86.77)
Hispanic	39,935 (13.3)
Education	
No high school diploma	4572 (1.5)
High school diploma	41,038 (13.6)
College and greater	255,210 (84.8)
Income	
Less than USD 25,000	47,395 (15.8)
USD 25,000 to USD 50,000	48,582 (16.1)
USD 50,000 to USD 100,000	75,463 (25.1)
USD 100,000 to USD 200,000	65,088 (21.6)
More than USD 200,000	64,292 (21.4)
Insurance	
Yes	286,233 (95.2)
No	14,587 (4.8)
Employment	
Yes	133,281 (44.3)
No	167,539 (55.7)
Marriage	
Yes	156,337 (52.0)
No	144,483 (48.0)

**Table 2:** Adjusted odds ratios (95% CI) for sociodemographic factors associated with overall DMC.

Covariates	OR [95% CI]	*p*-Values
Race [reference = White]		
Black	0.90 [0.88–0.93]	<0.01
Asian	0.83 [0.79–0.87]	<0.01
Others	1.17 [1.14–1.20]	<0.01
Ethnicity [reference: Non-Hispanic]		
Hispanic	0.86 [0.84–0.89]	<0.01
Gender [reference = Male]		
Female	1.44 [1.42–1.47]	<0.01
Income [Reference ≤ 25 k]		
25 k–50 k	0.91 [0.89–0.94]	<0.01
50 k–100 k	0.66 [0.64–0.68]	<0.01
100 k–200 k	0.48 [0.47–0.50]	<0.01
More than 200 k	0.49 [0.47–0.50]	<0.01
Employment [reference = unemployed]		
Employed	1.00 [0.98–1.02]	0.864
Education [reference = no high school diploma]		
High school diploma	1.06 [0.99–1.13]	0.098
College and above	1.04 [0.98–1.11]	0.222
Marriage [reference = not married]		
Married	0.83 [0.81–0.84]	<0.01
Insurance [reference = not insured]		
Insured	0.61 [0.59–0.63]	<0.01
Age [reference ≤ 40}		
Age (40–64)	0.56 [0.55–0.57]	<0.01
Age (65–74)	0.19 [0.18–0.19]	<0.01
Age (75–84)	0.13 [0.12–0.14]	<0.01
Age (≥85)	0.12 [0.10–0.13]	<0.01

Note: Values are adjusted odds ratios (AOR) with 95% CI.

## Data Availability

The original data presented in the study are not openly available. Restrictions apply to the availability of these data. Data were obtained from the All of Us Research Program and are available through the All of Us Researcher Workbench with permission of the All of Us Research Program after completion of required registration and training (https://www.researchallofus.org Accessed on 31 January 2026).

## Supplementary Materials

The following supporting information can be downloaded at: https://www.mdpi.com/article/10.3390/nursrep16020051/s1, Table S1: Adjusted odds ratios (95% CI) for sociodemographic predictors of delayed medical care, overall and by reason.

## Public Involvement Statement

No public involvement in any aspect of this research.

## Guidelines and Standards Statement

This manuscript was drafted in accordance with the Strengthening the Reporting of Observational Studies in Epidemiology (STROBE) Statement, which provides reporting guidelines for observational research, including cross-sectional, cohort, and case–control studies [32].

## Abbreviations

The following abbreviations are used in this manuscript:

AoU	All of Us
DMC	Delayed medical care
NIH	National Institute of Health
OR	Odds ratio
U.S.	United States
