# Supplementary material for: Sociodemographic Drivers of Delays in Seeking Medical Care in the All of Us Cohort
Source: Nurs Rep. 2026 Feb 2;16(2):51. doi: 10.3390/nursrep16020051 (PMC12943032; doi:10.3390/nursrep16020051)
Supplement: Supplementary file 1 [file nursrep-16-00051-s001.zip › nursrep-4104299-supplementary.pdf]

Covariates	Childcare	Copay	Deductible	Distance	Elderly care	Nervousness	OOP	Transportation	Work
Race [reference=White]									
Black	1.32 [1.22–1.42] **	1.03 [0.99–1.08]	0.89 [0.85–0.93] **	0.85 [0.80–0.90] **	1.10 [1.01–1.20] **	0.63 [0.61–0.66] **	0.81 [0.78–0.84] **	1.42 [1.36–1.48] **	1.01 [0.96–1.05]
Asian	0.73 [0.65–0.83] **	0.69 [0.64–0.75] **	0.77 [0.72–0.83] **	0.41 [0.35–0.48] **	1.05 [0.89–1.22]	0.61 [0.57–0.64] **	0.94 [0.89–0.99] *	0.87 [0.80–0.95] **	1.18 [1.11–1.25] **
Others	1.29 [1.21–1.38] **	1.20 [1.15–1.26] **	1.09 [1.04–1.13] **	1.30 [1.22–1.38] **	1.41 [1.30–1.52] **	0.96 [0.93–1.00] *	1.06 [1.03–1.10] **	1.43 [1.37–1.49] **	1.24 [1.19–1.29] **
Ethnicity [reference: Non-Hispanic]									
Hispanic	1.10 [1.02–1.18] **	0.86 [0.82–0.90] **	0.85 [0.81–0.89] **	0.66 [0.61–0.70] **	1.09 [1.00–1.20] *	0.77 [0.74–0.80] **	0.82 [0.79–0.86] **	0.82 [0.78–0.86] **	1.04 [0.99–1.08]
Gender [reference=Male]									
Female	2.50 [2.36–2.65] **	1.43 [1.39–1.48] **	1.33 [1.29–1.36] **	1.22 [1.17–1.27] **	1.64 [1.54–1.74] **	1.53 [1.50–1.57] **	1.31 [1.28–1.33] **	1.13 [1.09–1.16] **	1.37 [1.33–1.41] **
Income [Reference=<25k]									
25k-50k	0.97 [0.91–1.04]	1.22 [1.18–1.27] **	1.52 [1.46–1.58] **	0.67 [0.63–0.70] **	0.83 [0.77–0.90] **	0.90 [0.86–0.93] **	1.33 [1.29–1.38] **	0.55 [0.53–0.57] **	1.26 [1.20–1.31] **
50k-100k	0.77 [0.71–0.82] **	0.70 [0.67–0.73] **	1.11 [1.06–1.15] **	0.43 [0.40–0.45] **	0.56 [0.52–0.61] **	0.77 [0.75–0.80] **	1.03 [0.99–1.06]	0.33 [0.31–0.34] **	0.94 [0.91–0.98] **
100k-200k	0.67 [0.62–0.72] **	0.26 [0.25–0.28] **	0.60 [0.57–0.63] **	0.27 [0.25–0.29] **	0.41 [0.37–0.45] **	0.66 [0.63–0.68] **	0.70 [0.67–0.73] **	0.23 [0.22–0.24] **	0.73 [0.69–0.76] **
More than 200k	0.73 [0.68–0.79] **	0.38 [0.36–0.40] **	0.54 [0.51–0.56] **	0.43 [0.41–0.46] **	0.59 [0.54–0.64] **	0.61 [0.58–0.63] **	0.61 [0.58–0.63] **	0.43 [0.42–0.45] **	0.73 [0.70–0.76] **
Employment [reference=unemployed]									
Employed	0.66 [0.63–0.69] **	1.05 [1.02–1.08] **	1.18 [1.15–1.21] **	0.53 [0.50–0.55] **	0.64 [0.60–0.67] **	0.81 [0.79–0.83] **	1.06 [1.04–1.08] **	0.49 [0.47–0.50] **	2.82 [2.74–2.91] **
Education [reference=no high school diploma]									

High school diploma	0.81 [0.70–0.94] **	0.90 [0.82–0.99] *	0.95 [0.86–1.06]	1.03 [0.91–1.17]	1.20 [1.02–1.43] *	1.18 [1.07–1.31] **	1.27 [1.15–1.39] **	1.02 [0.93–1.11]	0.87 [0.78–0.98] **
College and above	0.62 [0.54–0.72] **	0.91 [0.82–1.00] *	1.07 [0.96–1.19]	0.67 [0.59–0.75] **	1.00 [0.85–1.18]	1.16 [1.05–1.29] **	1.66 [1.51–1.82] **	0.68 [0.63–0.74] **	0.98 [0.88–1.09]
Marriage [reference= not married]									
Married	2.45 [2.33–2.56] **	0.85 [0.82–0.87] **	1.00 [0.97–1.03]	0.92 [0.88–0.96] **	0.97 [0.92–1.03]	0.79 [0.77–0.81] **	0.93 [0.91–0.95] **	0.57 [0.55–0.59] **	0.86 [0.83–0.88] **
Insurance [reference=not insured]									
Insured	1.06 [0.97–1.15]	0.34 [0.33–0.36] **	0.45 [0.43–0.47] **	0.87 [0.82–0.94] **	0.87 [0.79–0.95] **	1.15 [1.09–1.20] **	0.34 [0.33–0.36] **	0.95 [0.90–1.00] **	0.85 [0.81–0.90] **
Age [reference=<40}									
Age (40-64)	0.22 [0.21–0.23] **	0.72 [0.70–0.74] **	0.82 [0.80–0.84] **	0.83 [0.79–0.86] **	1.61 [1.51–1.72] **	0.47 [0.46–0.48] **	0.79 [0.77–0.81] **	0.79 [0.77–0.82] **	0.47 [0.46–0.48] **
Age (65-74)	0.02 [0.01–0.02] **	0.20 [0.18–0.21] **	0.18 [0.17–0.19] **	0.32 [0.29–0.34] **	0.86 [0.78–0.94] **	0.17 [0.16–0.18] **	0.30 [0.29–0.31] **	0.33 [0.31–0.34] **	0.08 [0.08–0.09] **
Age (75-84)	0.01 [0.00–0.01] **	0.10 [0.09–0.11] **	0.08 [0.07–0.09] **	0.24 [0.21–0.27] **	0.64 [0.56–0.74] **	0.10 [0.09–0.10] **	0.23 [0.21–0.24] **	0.25 [0.23–0.27] **	0.02 [0.02–0.03] **
Age (≥85)	0.01 [0.00–0.03] **	0.06 [0.04–0.08] **	0.05 [0.03–0.08] **	0.14 [0.09–0.20] **	0.68 [0.47–0.95] *	0.06 [0.04–0.07] **	0.22 [0.19–0.26] **	0.27 [0.22–0.33] **	0.02 [0.01–0.04] **

Note: Values are adjusted odds ratios (AOR) with 95% CI; OOP=Out of pocket; ** =significant at 0.01; *= significant at 0.05 ; no asterisks:= no statistical significant association
